# Association of height, BMI, and smoking status with prostate cancer risk before and after the introduction of PSA testing in Sweden

**DOI:** 10.1038/s41598-025-06548-y

**Published:** 2025-06-25

**Authors:** Innocent B. Mboya, Josef Fritz, Pietro Scilipoti, Christel Häggström, Marisa da Silva, Ming Sun, Jens Wahlström, Viktor Oskarsson, Karl Michaëlsson, Jerzy Leppert, Abbas Chabok, Patrik K. E. Magnusson, Ylva Trolle Lagerros, Stephanie E. Bonn, Linnea Hedman, Pär Stattin, Tanja Stocks

**Affiliations:** 1 Department of Translational Medicine, Lund University, Malmö, Sweden; 2 Africa Academy for Public Health (AAPH), Dar es Salaam, Tanzania; 3 Department of Epidemiology and Biostatistics, School of Public Health, KCMC University, Moshi, Tanzania; 4 Institute of Clinical Epidemiology, Public Health, Health Economics, Medical Statistics and Informatics, Medical University of Innsbruck, Innsbruck, Austria; 5 Division of Experimental Oncology/Unit of Urology, URI Institution, IRCCS San Raffaele Hospital, Milan, Italy; 6 Department of Surgical Sciences, Uppsala University, Uppsala, Sweden; 7 Department of Diagnostics and Intervention, Northern Registry Centre, Umeå University, Umeå, Sweden; 8 School of Information Technology, Halmstad University, Halmstad, Sweden; 9 Department of Epidemiology and Global Health, Umeå University, Umeå, Sweden; 10 Department of Public Health and Clinical Medicine, Piteå Research Unit, Umeå University, Piteå, Sweden; 11 Medical Epidemiology, Department of Surgical Sciences, Uppsala University, Uppsala, Sweden; 12 Center for Clinical Research, Region Västmanland, Uppsala University, Uppsala, Sweden; 13 Department of Medical Epidemiology and Biostatistics, Karolinska Institutet, Stockholm, Sweden; 14 Division of Clinical Epidemiology, Department of Medicine, Karolinska Institutet, Solna, Stockholm, Sweden; 15 Center for Obesity, Academic Specialist Center, Stockholm Health Services, Stockholm, Sweden; 16 Department of Public Health and Clinical Medicine, The OLIN Unit, Umeå University, Umeå, Sweden

**Keywords:** Body mass index, Body height, Smoking, Prostate-specific antigen, Prostatic neoplasms., Cancer epidemiology, Cancer prevention, Cancer screening, Prostate cancer, Risk factors

## Abstract

Prostate cancer (PCa) incidence has steadily increased in Sweden, more steeply in the mid-1990s caused by increased opportunistic prostate-specific antigen (PSA) testing. Tallness, normal weight, and non-smoking are associated with more PSA testing, which increases detection of low-risk and localised PCa. We investigated time trends of height, body mass index (BMI), and smoking with PCa risk in 171,889 men in Sweden aged 50–64 years at baseline, who were linked to nationwide cancer registers during follow-up. Cox regression determined the association of these factors assessed before 1980, 1980–1994, and 1995–2004 with PCa risk. During 15 follow-up years, 8,049 men were diagnosed with PCa. The association of height with PCa was weakly positive across all calendar periods. For obesity (BMI ≥30 kg/m^2^) vs. normal weight (BMI 18.5–24.9 kg/m^2^) and current vs. never smoking, the associations changed from null before 1980 (HR 1.03, 95% CI 0.86–1.23, and 1.11, 95% CI 0.97–1.27) to negative in 1995–2004 (HR 0.83, 95% CI 0.74–0.93, and 0.86, 95% CI 0.79–0.93; p_interaction_ between periods = 0.05 and 0.001). In men with clinical characteristics available, height was positively associated with both aggressive and non-aggressive PCa whilst obesity and smoking showed negative associations only with non-aggressive PCa. These findings likely reflect differences in PSA testing by BMI and smoking habits and contribute important knowledge for etiological studies of PCa.

**Supplementary Information:**

The online version contains supplementary material available at 10.1038/s41598-025-06548-y.

## Introduction

Prostate cancer (PCa) is the most common cancer among men in high-income countries^1^. PCa incidence increased drastically with the introduction of prostate-specific antigen (PSA) testing in the 1990s^1,2^. For example, in Sweden, PCa incidence increased steadily since the 1960 s, with a steep rise between 1997 and 2004 when the PSA test was introduced and became widely used, and it has been quite stable since then^3^. The trend in Sweden is similar to the patterns in other Nordic countries^4^. PSA testing increases the detection of low-risk and localised PCa^5,6^. High uptake of PSA testing is associated with high socioeconomic status, taller stature and better health and lifestyle behaviours, including normal weight (body mass index [BMI] 18.5–24.9 kg/m^2^) and non-smoking^7–9^. The factors associated with a high uptake of PSA testing will also be associated with PCa diagnosis, irrespective of an actual biological link between them and PCa risk.

We and others have previously observed a negative association between obesity and smoking with PCa risk^6,8,10–12^. In the PSA era, that is after 1997, we have found negative associations between obesity, smoking, and localised or non-aggressive PCa, stronger negative associations for localised low-risk PCa, and no association with aggressive PCa^6,12^. Height has shown a weak positive association with PCa risk^6,8^, which may be biologically related to high exposure to insulin-like growth factor-1 (IGF-1) during adolescence. IGF-1 has, in turn, been associated with PCa risk, in particular with aggressive PCa^8^. At the same time, taller height is associated with the uptake of PSA testing, potentially due to its positive association with higher socioeconomic status^13,14^.

To further understand the effect that increased opportunistic PSA testing has had on the associations of height, BMI, and smoking with PCa risk, we investigated the associations of these factors with PCa risk, before and after the introduction of PSA testing in Sweden. We first confirmed data on these factors and the risk of aggressive and non-aggressive PCa, and then, investigated time trends from 1963 to 2019 of these factors on PCa risk.

## Materials and methods

### Study population and register linkages

The study used pooled nationwide data from the Obesity and Disease Development Sweden (ODDS) study, consisting of 4.3 million individuals, of which 2,164,945 were men, from Swedish cohorts and national registers with information on objectively measured or self-reported height and weight^15^. The self-reported height and weight was reported as current or recalled/historical. Information on smoking status was additionally collected in some of the cohorts. Using the unique personal identity number in Sweden, all individuals in the ODDS study were linked to national registers, including the Swedish Cancer Register, covering the whole population of Sweden since 1958 and capturing over 95% of all cancer diagnoses^16^. We also linked the population to the National Prostate Cancer Register (NPCR) to obtain information on cancer characteristics in PCa cases. The NPCR has been nationwide since the beginning of 1998^17^ and records detailed data on diagnostic cancer characteristics for 98% of all PCa cases compared to the Swedish Cancer Register^18^. We further retrieved information on the date of death from the Cause of Death Register^19^; sex, date of birth and emigration, country of birth, and marital status from the Total Population Register^20^; and education level from the Longitudinal integrated database for health insurance and labour market studies (LISA)^21^ and from the Population and housing censuses (in the 1960 s and 1970 s)^17^.

## Study sample

**Fig. 1:**
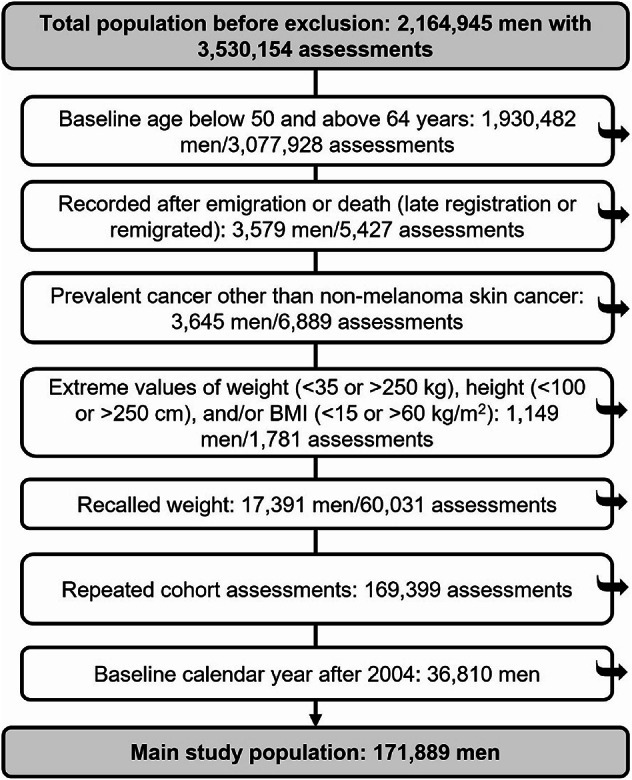
Flow diagram of individuals included in the study.

The study sample consisted of men in the ODDS study aged 50–64 years. This age range includes the ages at which men have been eligible for asymptomatic opportunistic PSA testing in Sweden^22,23^. The primary analysis of these men was performed in three subgroups of baseline calendar year (<1980, 1980–1994, 1995–2004) on PCa risk during a follow-up period of 15 years. Therefore, from the 3,530,154 records of body size in the 2,164,945 men in ODDS, we excluded records with a baseline age below 50 or above 64 years, date of entry recorded after emigration or death date due to late registration or remigration; prevalent cancers other than non-melanoma skin cancer; self-reported recalled weight (retaining self-reported current weight records in the study); extreme values of weight, height, and BMI; and repeated weight assessments. Assessments after 2004 were further excluded to allow for 15 years of follow-up until PCa diagnosis, death, or end of follow-up on 31 December 2019. The restrictions on baseline age and follow-up years ensured that these factors, and inherently also age at PCa diagnosis, were accounted for in time-trend analyses. After these exclusions, 171,889 men remained in the study (Fig. 1). Almost two-thirds originated from the Swedish Construction Workers Cohort (*n* = 98,898, 58%)^24^ and the rest came from over 10 other cohorts (Supplementary Table S1), the majority of which are population-based^15^.

## Main outcomes

The outcome of interest was PCa diagnosis (International Classification of Diseases seventh edition code 177) between 1963 and 2019 recorded in the Swedish Cancer Register^18^. PCa cases recorded in the NPCR diagnosed in 1998 or later, were categorised as aggressive in the presence of T4 or N1 or M1 or Gleason score ≥8 or a PSA level of ≥50 ng/mL^25^; other cases were categorised as non-aggressive.

## Ethics declarations

The ODDS study was approved by the Swedish Ethical Review Authority (no: 2020–03846 and 2024-07969-02). As also explained elsewhere^15^the study involves human participants and did not involve conducting any experiments. This study was performed according to the Declaration of Helsinki. Some of the included cohorts collected informed consent from participants, others did not. Informed consent from participants for the purpose of this study was waived by the Swedish Ethical Review Authority.

### Statistical analysis

Categorical variables were summarized using absolute frequencies and percentages. Normally distributed continuous variables were summarised using means with standard deviations, and non-normally distributed continuous variables using medians with interquartile range.

To investigate whether the study population reflected the general Swedish male population, we calculated age-standardised PCa incidence rates, directly standardised to the Swedish reference population of men aged 50–79 years at PCa diagnosis between 1963 and 2019. We obtained the reference population data from the Association of the Nordic Cancer Registries^26^ for men of the same age during the same calendar period.

To investigate the associations of height, BMI, and smoking with PCa risk, we used Cox regression with attained age as the time scale. We counted person-years (and events) from the date of baseline examination for a total of 15 years or until death, emigration, another cancer diagnosis, or PCa diagnosis, whichever occurred first. We estimated the hazard ratios (HRs) and 95% confidence intervals (CIs) of incident PCa according to height (per 5 cm, and quartiles [<172, 172 to <176, 176 to <180, ≥180 cm]), BMI (per 5 kg/m^2^, and in WHO categories [underweight, <18.5; normal weight, 18.5–24.9; overweight, 25.0–29.9; obesity; ≥30 kg/m^2^]), and smoking status (never, former, current).

To verify findings of previous studies of height, BMI, and smoking status with non-aggressive and aggressive PCa risk in our population^6,12^, we first investigated these associations (for 89,034 men). We counted person-years at risk from 1 January 1998 (the start of NPCR) or the date of the baseline examination, whichever came later, until death, emigration, another cancer diagnosis, or PCa diagnosis, whichever occurred first.

Time trend analyses were performed in periods of the baseline calendar year (<1980, 1980–1994, 1995–2004), which, considering the 15-year follow-up period, covered the timespans before, during, and after the introduction of PSA testing in Sweden. The Cox models were stratified by birth cohorts (<1920, 1920–1929, 1930–1939, ≥1940) and adjusted for education level (pre-upper secondary ≤9 years, upper secondary ≤3 years, post-upper secondary ≥ 3 years), marital status (unmarried, married/registered partner, divorced/widower/widower of partner), modes of height and weight assessment (measured, self-reported), birth country (participant and both parents born in Sweden, others), and smoking status (only for BMI analyses). Departures from the proportional hazards assumption were tested using Schoenfeld residuals and log-log survival plots for the primary exposures and covariates, with no major violations observed. We conducted tests for trends across categories of height quartiles, BMI categories, and smoking status categories, using the Wald test of linear associations, with categories treated as an ordinal variable, in the Cox regression models adjusted for covariates. P-values for the interaction of incident PCa HRs between the earliest (<1980) and latest calendar periods (1995–2004) for obesity versus normal weight, highest versus lowest height quartiles, continuous height and BMI, and current versus never smokers were calculated according to Altman and Bland^27^.

We used flexible parametric survival models to further investigate time trends of the associations of incident PCa with obesity vs. normal weight, highest vs. lowest height quartiles, and current vs. never smokers as a function of attained calendar year time scale. The models were adjusted for the same covariates as described above and additionally adjusted for the birth cohort. In contrast to the analysis of categorical baseline calendar year, which shows HRs for a broad range of baseline and attained calendar years, the results from the flexible parametric spline models show the HR continuously for the exact year of PCa diagnoses.

All statistical tests were evaluated using a significance level of 0.05. Data were analysed using Stata MP version 18.0 (StataCorp LLC, College Station, Texas, USA).

## Results

The 171,889 men in the study had a median baseline age (IQR) of 54 (51, 58) years, and it was similar across all calendar periods. Participant characteristics by categories of baseline calendar year are summarised in Table 1. Overall, men became taller over time, with the proportion in the highest height quartile (≥180 cm) doubling from 19% before 1980 to 39% in 1995–2004. Over the same period, obesity increased from 8 to 12%, and the prevalence of current smoking status decreased from 54 to 29%.

**Table 1:** Characteristics of study participants across calendar periods.

**Characteristics**	**Baseline year**			
	**<1980**	**1980–1994**	**1995–2004**	**Total**
N (% of total)	65,910 (38)	57,502 (33)	48,477 (28)	171,889 (100)
Baseline age, median (IQR)	55 (52, 59)	52 (51, 55)	55 (51, 60)	54 (51, 58)
Baseline year, median (IQR)	1974 (1972, 1975)	1987 (1983, 1991)	1997 (1997, 2000)	1984 (1975, 1997)
Birth year, median (IQR)	1918 (1914, 1922)	1933 (1930, 1938)	1943 (1939, 1946)	1931 (1921, 1940)
Follow-up time, median (IQR)	15.0 (14.5, 15.0)	15.0 (15.0, 15.0)	15.0 (15.0, 15.0)	15.0 (15.0, 15.0)
Weight (kg), median (IQR)	77.0 (70.0, 84.0)	80.0 (73.0, 87.0)	82.0 (75.0, 90.0)	79.0 (72.0, 87.0)
Height (cm), median (IQR)	174 (170, 178)	176 (172, 180)	178 (173, 182)	176 (172, 180)
BMI (kg/m^2^), median (IQR)	25.4 (23.5, 27.5)	25.7 (23.8, 27.8)	25.8 (23.9, 28.0)	25.6 (23.7, 27.7)
Height quartiles (cm), n (%)				
Q1; <172	21,278 (32)	12,821 (22)	7,654 (16)	41,753 (24)
Q2; 172 to <176	17,302 (26)	13,576 (24)	10,230 (21)	41,108 (24)
Q3; 176 to <180	14,670 (22)	14,055 (24)	11,502 (24)	40,227 (23)
Q4; ≥180	12,660 (19)	17,050 (30)	19,091 (39)	48,801 (28)
BMI category (kg/m^2^), n (%)				
<18.5	248 (<1)	219 (<1)	143 (<1)	610 (<1)
18.5–24.9	28,939 (44)	23,267 (40)	18,995 (39)	71,201 (41)
25.0–29.9	31,181 (47)	28,053 (49)	23,639 (49)	82,873 (48)
≥30	5,542 (8)	5,963 (10)	5,700 (12)	17,205 (10)
Smoking status, n (%)*				
Never	14,668 (33)	21,538 (38)	16,731 (35)	52,937 (36)
Former	5,871 (13)	17,214 (30)	16,838 (35)	39,923 (27)
Current	23,941 (54)	17,816 (31)	14,027 (29)	55,784 (38)
Birth year, n (%)				
<1920	36,985 (56)	228 (<1)	0 (0)	37,213 (22)
1920–1929	28,925 (44)	12,422 (22)	0 (0)	41,347 (24)
1930–1939	0 (0)	35,107 (61)	14,525 (30)	49,632 (29)
≥1940	0 (0)	9,745 (17)	33,952 (70)	43,697 (25)
Educational level, n (%)*^†^				
Pre-upper secondary school, ≤9 years	48,662 (82)	32,560 (57)	15,120 (31)	96,342 (58)
Upper secondary school, ≤3 years	10,077 (17)	20,626 (36)	21,168 (44)	51,871 (31)
Post-upper secondary school, ≥3 years	585 (1)	4,156 (7)	12,155 (25)	16,896 (10)
Marital status, n (%)				
Unmarried	6,169 (9)	5,354 (9)	6,073 (13)	17,596 (10)
Married/registered partner	54,721 (83)	43,900 (76)	34,599 (71)	133,220 (78)
Divorced/Widower	5,020 (8)	8,248 (14)	7,805 (16)	21,073 (12)
Height, n (%)^‡^				
Objectively measured	61,421 (93)	57,501 (>99)	26,338 (54)	145,260 (85)
Self-reported	4,489 (7)	1 (<1)	22,139 (46)	26,629 (15)
Weight, n (%)^‡^				
Objectively measured	61,187 (93)	57,502 (100)	17,907 (37)	136,596 (79)
Self-reported	4,723 (7)	0 (0)	30,570 (63)	35,293 (21)
Birth country for participant and parents, n (%)				
All born in Sweden	63,687 (97)	52,179 (91)	44,176 (91)	160,042 (93)
Other	2,223 (3)	5,323 (9)	4,301 (9)	11,847 (7)

* Number of men with missing values: smoking status, 23,245 (14%); education level, 6,780 (4%).

^**†**^ Regards to the highest attained education through follow-up.

^‡^ The high proportion with self-reported height and weight in the 1995–2004 period is due to the dominance of the Cohort of Swedish Men, in which body size was self-reported. There were no data in this period from the large Construction Workers Cohort, in which body size was objectively measured.

**Fig. 2:**
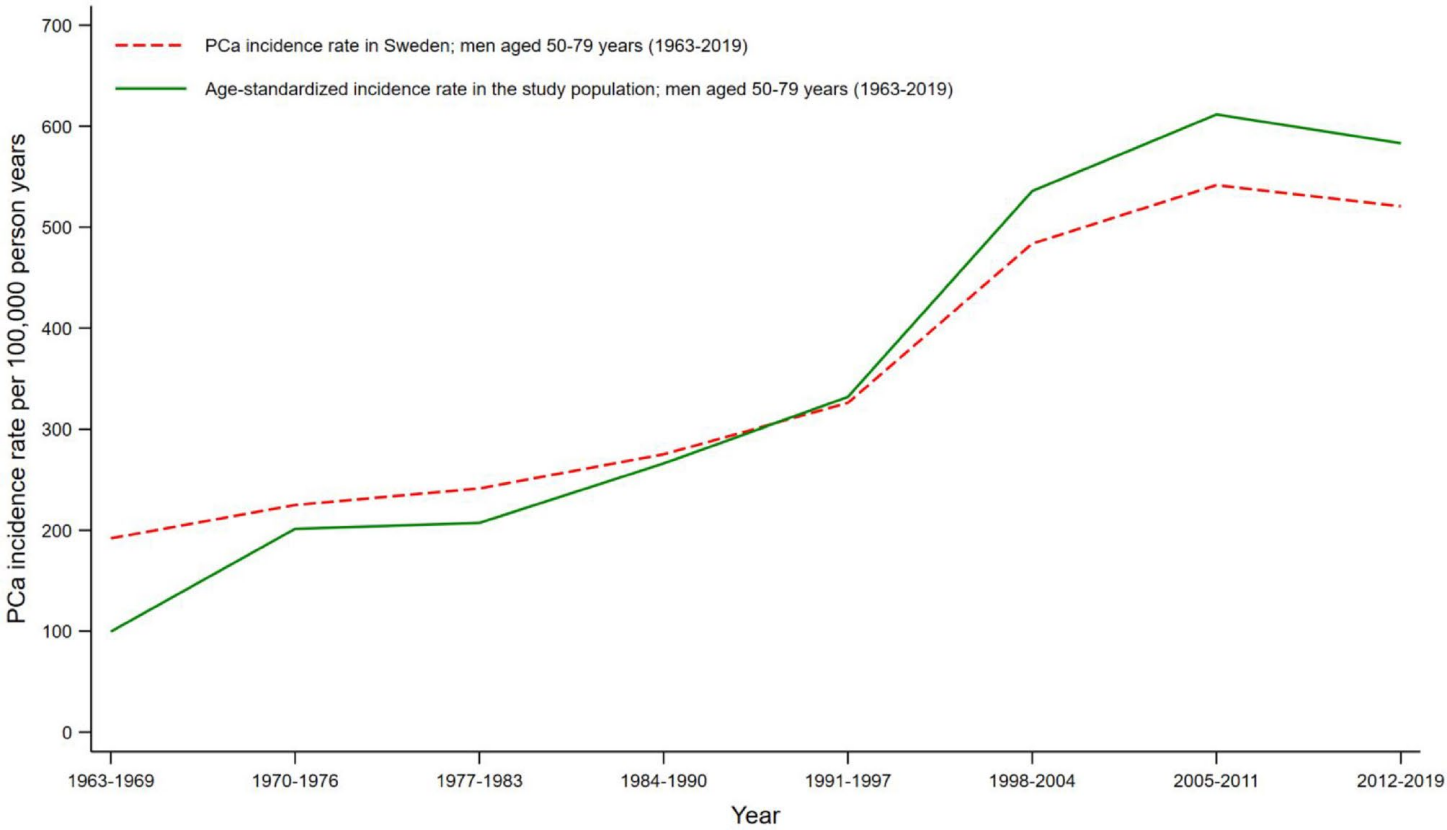
Prostate cancer incidence rates by calendar years. Prostate cancer incidence rate in Sweden (red dashed curve) obtained from the Association of the Nordic Cancer Registries for men aged 50–79 years between 1963 and 2019.^26^ The age-standardized incidence rate in the study population (green solid line) was standardized using the Swedish reference population for men aged 50–79 years between 1963 and 2019. Abbreviations: PCa, prostate cancer.

During 2,329,188 person-years of follow-up, 8,049 (5%) men were diagnosed with PCa. The mean age at PCa diagnosis was 66 (standard deviation 5) years. The age-standardised PCa incidence rate was 99.6 per 100,000 person-years in 1963-69, which increased steadily over time. The steepest increase was observed between the years 1991 and 2004 (age-standardised PCa incidence rate 535.8 per 100,000 person-years in 1998–2004), similar to the trend in the Swedish reference population (Fig. 2).

The shape of the association between height and BMI with total, non-aggressive, and aggressive PCa is shown in Supplementary Fig S1 and confirms the findings from our previous, larger study^6^. Height was positively associated with total, non-aggressive, and aggressive PCa up to a height of 175 cm, after which the association flattened out. BMI was positively associated with total PCa and non-aggressive PCa up to a BMI of 25 kg/m^2^, after which the association became negative. The positive associations of height with both non-aggressive and aggressive PCa and the negative association of obesity only with non-aggressive PCa were further visualised in analyses of height and BMI in categories. Furthermore, similar to BMI and the results of smoking and PCa risk in our previous larger study^12^, we observed a negative association between current smoking with non-aggressive but not with aggressive PCa (Table 2).

**Table 2:** Hazard ratios for total, non-aggressive, and aggressive prostate cancer risk according to height, body mass index, and smoking status.

Variables	Total PCa (*n* men = 171,889)		Non-aggressive PCa (*n* men = 89,034)		Aggressive PCa (*n* men = 89,034)	
	Cases (*n* = 8,049)	HR (95% CI)	Cases (*n* = 3,984)	HR (95% CI)	Cases (*n* = 1,181)	HR (95% CI)
Height quartiles (cm)						
Q1; <172	1,647	1.00	622	1.00	199	1.00
Q2; 172 to <176	1,939	1.12 (1.05, 1.19)	890	1.12 (1.01, 1.25)	277	1.13 (0.94, 1.36)
Q3; 176 to <180	1,922	1.09 (1.02, 1.17)	959	1.10 (0.99, 1.22)	296	1.13 (0.94, 1.36)
Q4; ≥180	2,541	1.10 (1.04, 1.18)	1,513	1.14 (1.04, 1.26)	409	1.07 (0.90, 1.27)
Per 5 cm	8,049	1.03 (1.01, 1.05)	3,984	1.03 (1.01, 1.06)	1,181	1.03 (0.98, 1.07)
*P-value* for trend		0.02		0.02		0.66
BMI category (kg/m^2^)						
<18.5	22	0.91 (0.60, 1.38)	9	0.87 (0.45, 1.68)	5	1.62 (0.67, 3.91)
18.5–24.9	3,237	1.00	1,622	1.00	444	1.00
25.0–29.9	4,048	1.04 (0.99, 1.09)	2,005	0.99 (0.92, 1.05)	599	1.06 (0.94, 1.20)
≥30	742	0.90 (0.83, 0.97)	348	0.75 (0.67, 0.85)	133	1.01 (0.83, 1.23)
Per 5 kg/m^2^	8,049	0.99 (0.95, 1.01)	3,984	0.90 (0.85. 0.94)	1,181	1.03 (0.95, 1.13)
*P-value* for trend		0.31		< 0.001		0.72
Smoking status*						
Never	2,671	1.00	1,518	1.00	441	1.00
Former	2,239	0.99 (0.94, 1.05)	1,403	0.98 (0.91, 1.06)	402	0.98 (0.85, 1.12)
Current	2,257	0.95 (0.89, 1.00)	997	0.88 (0.81, 0.95)	317	0.95 (0.83, 1.11)
*P-value* for trend		0.08		0.002		0.53

The analyses of non-aggressive and aggressive PCa included 89,034 non-censored men by January 1, 1998, when the Swedish National Prostate Cancer Register became nationwide.

Hazard ratios were derived from Cox regression models with BMI, height, and smoking status modelled in categories with attained age as the time scale, stratified by birth cohort (< 1920, 1920–1929, 1930–1939, ≥ 1940). Tests for trend across categories of height quartiles, BMI, and smoking status, were conducted using the Wald test of linear associations, with categories treated as an ordinal variable, from the Cox regression models adjusted for covariates. Estimates were adjusted for education level, marital status, mode of height and weight assessment, birth country, and smoking status. Estimates for height were not adjusted for BMI and smoking status. Estimates for smoking status were not adjusted for BMI, height, and modes of height and weight measurement. * Smoking status analyses excluded the missing information; 23,245 (14%) for incident PCa and 1256 (1.4%) for non-aggressive and aggressive PCa analyses. Abbreviations: HR, Hazard Ratio. PCa, Prostate cancer. CI. Confidence Interval.

**Fig. 3:**
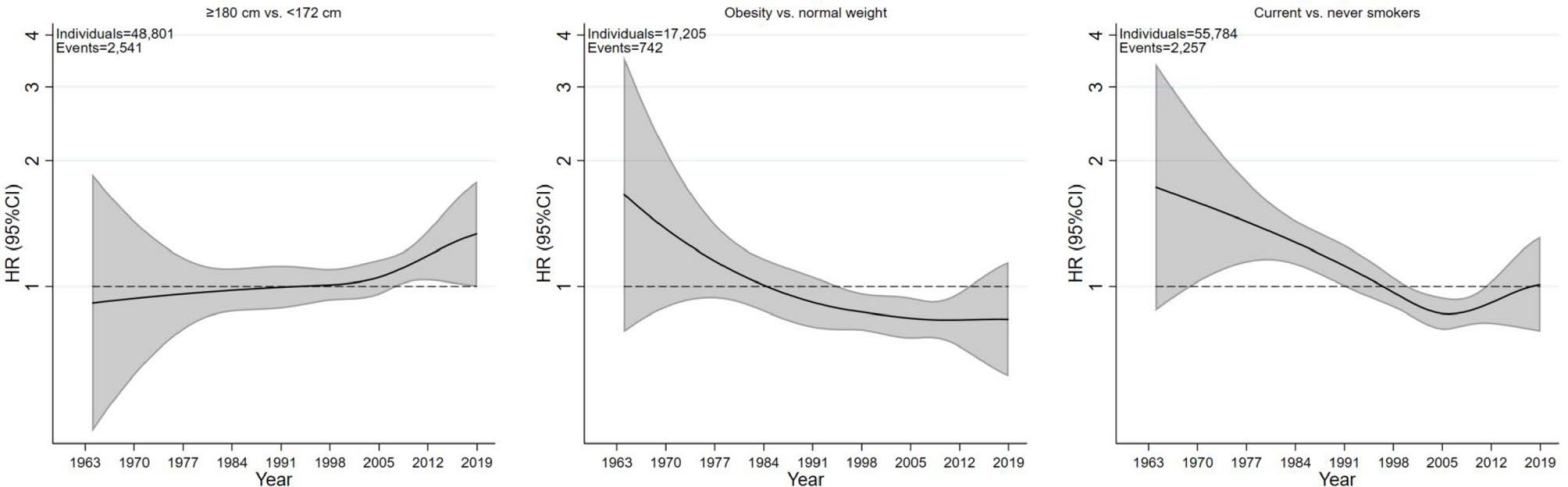
Hazard ratios for prostate cancer risk according to height, body mass index, and smoking status on attained calendar year time scale. Hazard ratios were derived from flexible parametric survival regression models with BMI, height, and smoking status modelled in categories on attained calendar year as the time scale. Solid lines are hazard ratio point estimates, and shaded areas indicate 95% confidence intervals. Estimates for BMI were adjusted for education level, marital status, mode of height and weight assessment, birth country, and birth year categories. Estimates for height were not adjusted for BMI and smoking status. Estimates for smoking status were not adjusted for BMI, height, and modes of height and weight measurement. P-value for trend: height (p=0.19), BMI (p=0.08), smoking status (p<0.001) – obtained using the Wald test of linear associations, with categories treated as an ordinal variable, from the flexible parametric survival regression models adjusted for covariates. HR, Hazard Ratio. CI. Confidence Interval.

The time trends of the association between height, BMI, and smoking status with PCa risk across baseline calendar periods are shown in Table 3. For height (≥180 vs. <172 cm), the association with PCa changed from a null association before 1980 (HR 1.04, 95% CI, 0.91–1.19) to a positive association in 1995–2004 (1.12, 95% CI, 1.02–1.24), but without formal evidence for a time trend (*p*_*interaction*_ between periods = 0.38). The lack of a time trend was even more evident in the analysis of height as a continuous variable (HR per 5 cm, 1.03 across all three periods, *p*_*interaction*_>0.99). For obesity and current smoking, the associations changed from null associations before 1980 (HR 1.03, 95% CI, 0.86–1.23, and 1.11, 95% CI, 0.97–1.27) to negative associations in 1995–2004 (HR 0.83, 95% CI, 0.74–0.93, and 0.86, 95% CI, 0.79–0.93), with evidence of time trend effects (*p*_*interaction*_ between periods = 0.05 and 0.001). Analyses using flexible parametric survival models with attained calendar year time scale showed slight increases in the HRs for PCa risk regarding tallness over time and with clear decreases in the HRs for PCa risk for obesity and current smoking over time, which, however, flattened out in the early 2000 s (Fig. 3).

**Table 3:** Hazard ratios for prostate cancer risk according to height, body mass index, and smoking status across baseline calendar year periods.

Variable	<1980 (*n* men = 65,910)		1980–1994 (*n* men = 57,502)		1995–2004 (*n* men = 48,477)		*P*-value for interaction between periods*
	Cases (*n* = 1,840)	HR (95% CI)	Cases (*n* = 2,304)	HR (95% CI)	Cases (*n* = 3,905)	HR (95% CI)	
Height quartiles (cm)							
Q1; <172	598	Ref (1.00)	466	Ref (1.00)	583	Ref (1.00)	
Q2; 172 to <176	496	1.07 (0.95, 1.21)	598	1.21 (1.07, 1.37)	845	1.09 (0.98, 1.21)	
Q3; 176 to <180	420	1.09 (0.96, 1.23)	548	1.07 (0.94, 1.21)	954	1.12 (1.01, 1.24)	
Q4; ≥180	326	1.04 (0.91, 1.19)	692	1.12 (0.99, 1.26)	1,523	1.12 (1.02, 1.24)	0.38
Per 5 cm	1,840	1.03 (0.99, 1.07)	2,304	1.03 (1.00, 1.07)	3,905	1.03 (1.01, 1.06)	> 0.99
*P-value* for trend		0.40		0.36		0.03	
BMI categories (kg/m^2^)							
<18.5	5	0.79 (0.33, 1.91)	9	1.17 (0.61, 2.27)	8	0.78 (0.39, 1.55)	
18.5–24.9	761	Ref (1.00)	894	Ref (1.00)	1,582	Ref (1.00)	
25.0–29.9	926	1.11 (1.01, 1.22)	1,183	1.06 (0.97, 1.16)	1,939	0.98 (0.92, 1.05)	
≥30	148	1.03 (0.86, 1.23)	218	0.90 (0.78, 1.05)	376	0.83 (0.74, 0.93)	0.05
Per 5 kg/m^2^	1,840	1.06 (0.98, 1.14)	2,304	0.99 (0.93, 1.05)	3,905	0.93 (0.89, 0.98)	0.004
*P-value* for trend		0.13		0.71		0.02	
Smoking status**							
Never	330	Ref (1.00)	864	Ref (1.00)	1,477	Ref (1.00)	
Former	114	0.91 (0.73, 1.13)	772	1.11 (1.01, 1.22)	1,353	0.91 (0.85, 0.98)	
Current	643	1.11 (0.97, 1.27)	610	1.00 (0.90, 1.11)	1,004	0.86 (0.79, 0.93)	0.001
*P-value* for trend		0.10		0.77		< 0.001	

Hazard ratios were derived from Cox regression models on attained age as time scale, stratified by birth cohorts (<1920, 1920–1929, 1930–1939, ≥1940). Tests for trend across categories of height quartiles, BMI, and smoking status, were conducted using the Wald test of linear associations, with categories treated as an ordinal variable, from the Cox regression models adjusted for covariates. Estimates were adjusted for education level, marital status, mode of height and weight assessment, birth country, and smoking status. Estimates for height were not adjusted for BMI and smoking status. Estimates for smoking status were not adjusted for BMI, height, and modes of height and weight measurement. *The p-values for interaction between calendar periods (<1980 vs. 1995–2004) were calculated as in Altman and Bland (2003).^27^ ** Smoking status analysis excluded men with missing smoking status information; 23,245 (14%). Abbreviations: BMI, Body mass index. HR, Hazard Ratio. CI. Confidence Interval.

## Discussion

In this study, we have examined the time trends of height, BMI and smoking status with PCa risk in Sweden, all of which are associated with the uptake of PSA testing^7^. The investigation originated from the steep increase in PCa incidence in the 1990 s in Sweden resulting from the introduction of opportunistic PSA testing^3,4^, which could affect PCa risk associations of factors also associated with PSA testing uptake. The associations of BMI (obesity vs. normal weight) and smoking status (current vs. never) with PCa risk changed from null to negative. We also found a negative association between obesity and current smoking only with non-aggressive PCa, typically PSA-detected, suggesting an effect of increased PSA testing on the association of BMI and smoking with PCa risk. By contrast, we found no evidence of a time trend in the association between height and PCa risk across the pre- and PSA testing era (the mid-1990s onwards).

A positive association between height and PCa risk has previously been reported in studies based on data in Sweden^6^, as well as in an umbrella review of prospective observational and Mendelian randomisation studies, albeit with weak evidence^8^. There is strong evidence of a positive association between height and socioeconomic status in men^13,14^, and PSA testing and PCa incidence are more common in men with high socioeconomic status^7,9^. Despite adjusting for different sociodemographic factors, that is education level, marital status, and birth country, the positive associations between height and PCa risk in this study persisted across calendar periods, but could still be affected by residual confounding by socioeconomics. It is possible that a positive association with non-aggressive PCa could primarily be driven by more PSA testing in tall men, whereas a positive association with aggressive PCa could primarily be caused by biological effects related to height^7^. IGF-1, a growth factor related to tallness through its association with prepubertal growth^8,28^, has been implicated in the aetiology of high-risk and advanced PCa^8^. Height could also be a marker of genetic, environmental, hormonal, and nutritional factors affecting both growth and PCa risk^29^. The similarities in the association of height with non-aggressive and aggressive PCa are likely why we did not observe a time trend in the association between height and PCa risk.

Obesity is negatively associated with overall and localised or non-aggressive PCa^6,8,30^. These types of PCa have increased over time in Sweden^31^, consistent with the increased uptake of PSA testing^4,32^. Our study showed that the association of BMI (obesity vs. normal weight) with PCa risk changed from a null association before the PSA era to a strong and negative association after PSA testing became available to most men, with evidence of interaction between calendar periods. Interestingly, the inverse U-shaped association of BMI with PCa risk, as observed in our and other studies^6,30^, is consistent with the association of BMI with PSA testing activity; normal to overweight men undergo PSA testing the most, while both men with underweight and obesity are screened less, thereby delaying or avoiding a PCa diagnosis^7,33^. Taken together, the introduction of PSA testing in Sweden is likely to have influenced our observed time trends of BMI with PCa risk. An overall null association before the PSA era and a null association with aggressive PCa in the PSA era, as demonstrated in various Mendelian randomisation studies^34,35^, suggest no biological association of obesity with incident PCa.

The association between smoking status (current vs. never) and risk of PCa also changed from null before the PSA era to strong and negative in the PSA era, with evidence of interaction between periods. Several systematic reviews, meta-analyses, and Mendelian randomisation studies provide strong evidence of a negative association between current smoking and PCa risk, especially localised/non-aggressive PCa^8,10,11,36^. This association is mostly observed in studies conducted during the PSA era^10,12,36^. Additionally, our previous study using five Swedish cohorts showed that smoking in combination with obesity was associated with a further decrease in risk of non-aggressive PCa^12^. Similar to men with obesity, smokers are less likely to take an asymptomatic PSA test^37^, which together with the increased availability of such tests over time, most likely explain our observed time trend of the association between smoking and PCa.

Our study has limitations, one being changes over time in the definition of the variables, especially smoking status, due to different questionnaires used in the included cohorts. Another limitation is that the inclusion of self-reported weight and height to some extent may result in either under- or over-estimation of their magnitude, even though the correlation with objectively measured weight and height is generally high^38,39^. A direct comparison between self-reported and objectively measured body size was not possible in our data, given that these men also differed by cohort, age, and calendar year. Furthermore, we lacked direct information regarding PSA testing activity, including the characteristics of men who underwent testing and the timing of their tests. However, despite the lack of a national PCa screening program, it should be noted that around 40 to 60% of men in Sweden’s largest county, Stockholm, aged between 50 and 69 years, had taken a PSA test during the last five years in 2011^40^.

Strengths of our study include the large sample size, which allowed us to examine time trends between height, BMI, smoking, and PCa risk, accounting for important characteristics including baseline age and follow-up time. The high coverage and validity of Swedish registers is a further strength^16,18,20^. The fact that the trends of PCa incidence in Sweden were similar to the trends in our data supports the assumption that our study population was representative of the full Swedish male population. The replication of exposure-outcome associations in previous, larger studies of a Swedish population^6,12^, also supports high external validity.

Furthermore, findings from this study can most likely be generalized to similar settings of opportunistic testing or organised screening of cancer, which imply similar incidence shifts. For example, in Sweden, men who have attended PCa screening in clinical trials and women attending breast cancer screening have higher socioeconomic status than non-attendants^22,41^. Similar to the patterns for PSA testing in men^7^, women undertaking breast cancer screening generally have healthier lifestyles than non-attendants^42^. Such differences according to attendance together with any differences in associations between a screen-related factor and screen-detected vs. symptomatic cancer (as for BMI and smoking with non-aggressive vs. aggressive PCa in our study) should always be reason for cautious interpretation of the factor’s potential biological association with a specific cancer.

## Conclusion

In this study, the association between obesity, smoking, and PCa risk in Sweden changed from null before the PSA era to negative strong associations when PSA testing became available to most men. While these factors and height are associated with PSA testing behaviour, only obesity and current smoking are differentially associated with PCa risk by tumour aggressiveness, specifically by having a negative association with non-aggressive/localised PCa, which is typically PSA-detected. Taken together, the observed time trends of this study likely reflect differences in PSA testing by BMI and smoking habits and contribute important knowledge for etiological studies of PCa.

## Electronic supplementary material

Below is the link to the electronic supplementary material.

Supplementary Material 1

## Data Availability

All data are located on Statistics Sweden’s Microdata Online Access (MONA) server and may only be accessed from countries in the European Union or the European Economic Area. Data access covered by ethical approval will be considered in agreement with the principal investigator of ODDS, Tanja Stocks (tanja.stocks@med.lu.se), and upon approval from register holders and steering committees of ODDS cohorts.
