# Supplementary material for: Association of height, BMI, and smoking status with prostate cancer risk before and after the introduction of PSA testing in Sweden
Source: Sci Rep. 2025 Jun 25;15:20290. doi: 10.1038/s41598-025-06548-y (PMC12198391; doi:10.1038/s41598-025-06548-y)
Supplement: Supplementary file 1 — Supplementary Material 1 [file 41598_2025_6548_MOESM1_ESM.docx]

**Association of height, BMI, and smoking status with prostate cancer risk, before and after the introduction of** **opportunistic PSA testing in Sweden**

# Table S1: Number of men in the final study sample for each cohort, overall and by incident prostate cancer cases and non-cases.

| Cohort (Year of Baseline Examination) | Non-cases | Incident PCa cases | Total |
| --- | --- | --- | --- |
| Swedish Construction Workers Cohort, (1971-1993) | 95,943 | 2,955 | 98,898 |
| Malmö Diet and Cancer Study (MDCS), (1991-2000) | 5,934 | 502 | 6,436 |
| Malmö Preventive Project (MPP), (1976-1991) | 6,017 | 246 | 6,263 |
| National March Cohort, (1997) | 3,407 | 358 | 3,765 |
| Monitoring of Trends of Cardiovascular Disease Study in Northern Sweden (MONICA), (1986-2004) | 1,368 | 88 | 1,456 |
| Västerbotten Intervention Programme (VIP), (1985-2004) | 16,592 | 1,310 | 17,902 |
| Obstructive Lung Disease in Norrbotten (OLIN), (1986-2004) | 1,191 | 59 | 1,250 |
| Cohort of Swedish Men (COSM), (1997) | 15,698 | 1,418 | 17,116 |
| Q63, 67, 70, (1963-1970) | 4,634 | 89 | 4,723 |
| Screening Across the Lifespan Twin Study (SALT), (1998-2004) | 8,063 | 793 | 8,856 |
| Westmannia Cardiovascular Risk Factors Study (WICTORY), (1990-2000) | 4,993 | 231 | 5,224 |
| Total (1963-2004) | 163,840 | 8,049 | 171,889 |

# Figure S1: Shape of association for height and body mass index with total, non-aggressive, and aggressive prostate cancer.


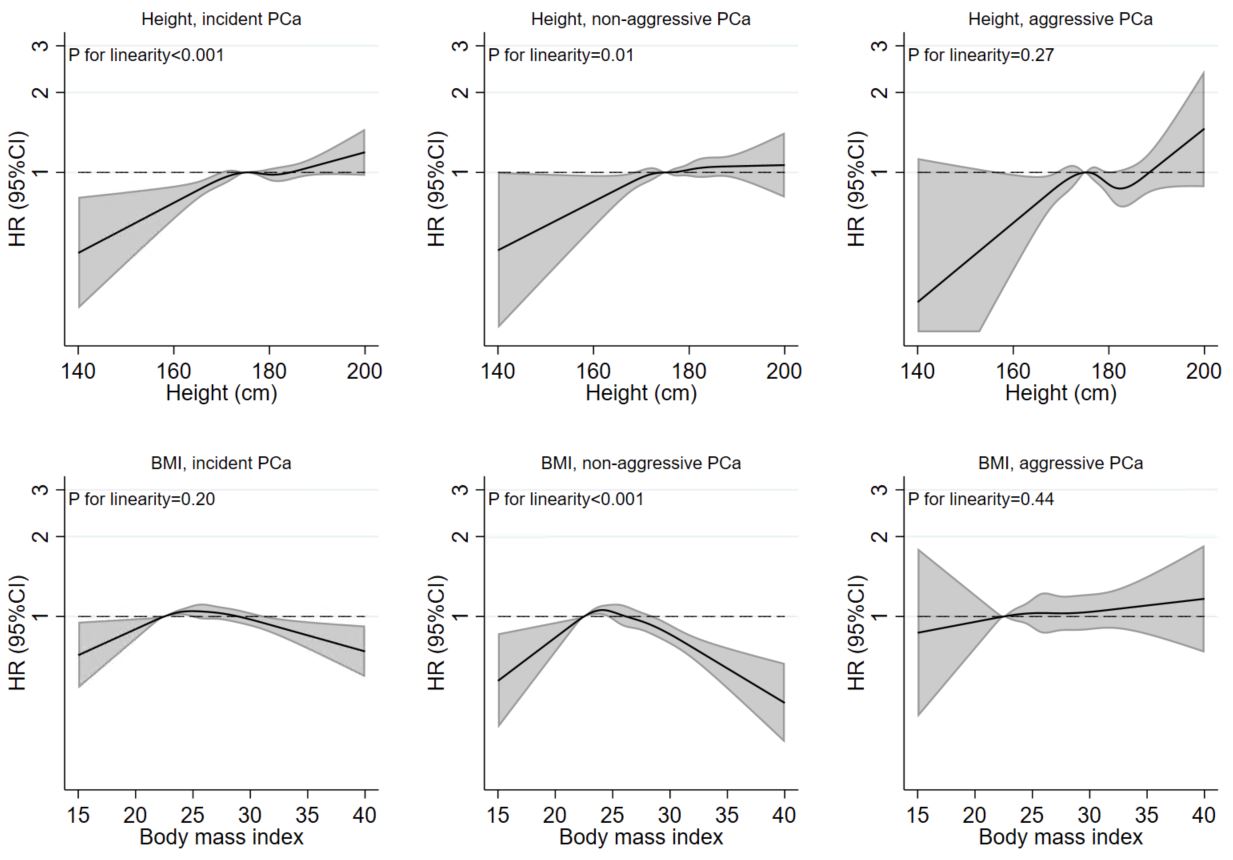


Note: The analyses of non-aggressive and aggressive PCa included 89,034 non-censored men by January 1, 1998, when the Swedish National Prostate Cancer Register became nationwide. Hazard ratios were derived from Cox regression models with BMI and height modelled using restricted cubic splines on attained age as the time scale, stratified by birth cohorts (<1920, 1920-1929, 1930-1939, ≥1940). Solid lines are hazard ratio point estimates, and shaded areas indicate 95% confidence intervals. Splines for height are scaled to range between 140 to 200 cm. Estimates were adjusted for education level, marital status, mode of height and weight assessment, birth country, and smoking status (in the analysis of BMI as exposure). P for linearity obtained from similar Cox models of continuous height and BMI separately for each event.

Abbreviations: HR, Hazard Ratio. PCa, Prostate cancer. CI. Confidence Interval.
